# Counseling and support services for healthcare workers in German university hospitals during the pandemic—descriptive results of a Germany-wide cross-sectional survey

**DOI:** 10.3389/fpubh.2023.1186929

**Published:** 2023-08-10

**Authors:** Elisabeth Diehl, Lina Marie Mülder, Carolin Imm, Peter Kegel, Marian Tolksdorf, Hauke Felix Wiegand, Nikolaus Röthke, Oliver Tüscher, Klaus Lieb, Henrik Walter, Susanne Liebe, Birgit Maicher, Sabine Hellwig, Kristina Adorjan, Stefan Unterecker, Manfred Beutel, Dirk-Matthias Rose

**Affiliations:** ^1^Institute of Occupational, Social and Environmental Medicine, University Medical Centre, Johannes Gutenberg University Mainz, Mainz, Germany; ^2^Department of Psychosomatic Medicine and Psychotherapy, University Medical Centre, Johannes Gutenberg University Mainz, Mainz, Germany; ^3^Department of Work, Organizational and Business Psychology, Institute of Psychology, Johannes Gutenberg University Mainz, Mainz, Germany; ^4^Department of Psychiatry and Psychotherapy, University Medical Centre, Johannes Gutenberg University Mainz, Mainz, Germany; ^5^Leibniz Institute of Resilience (LIR), Mainz, Germany; ^6^Department of Psychiatry and Psychotherapy, Charité-Universitätsmedizin Berlin, Corporate Member of Freie Universität Berlin and Humboldt-Universität zu Berlin, Berlin, Germany; ^7^Department of Occupational Health and Safety, Carl Gustav Carus University Hospital, Dresden, Germany; ^8^Department of Psychiatry and Psychotherapy, Faculty of Medicine, Carl Gustav Carus University Hospital, Dresden, Germany; ^9^Department of Psychiatry and Psychotherapy, Faculty of Medicine, University Medical Centre Freiburg, University of Freiburg, Freiburg, Germany; ^10^Department of Psychiatry and Psychotherapy, University Hospital, Ludwig Maximilian University of Munich, Munich, Germany; ^11^Department of Psychiatry, Psychosomatics and Psychotherapy, University Hospital Würzburg, Würzburg, Germany

**Keywords:** SARS-CoV-2 pandemic, COVID, corona, frontline healthcare worker, second-line healthcare worker, employee assistance program (EAP), counseling services, support services

## Abstract

**Background:**

Due to the SARS-CoV-2 pandemic, healthcare workers (HCWs) are experiencing tremendous levels of emotional and physical stress. Hospitals are trying to help personnel cope with work-related pressure. The aim of this study was to assess HCWs’ awareness and utilization of counseling and support services during the pandemic, HCWs’ unmet counseling and support needs, and the type and content of these services.

**Methods:**

A cross-sectional online survey was conducted from January to June 2021 through the German national research organization Network University Medicine (NUM). All participating hospitals (6 in total) were asked to inform their employees about the study.

**Results:**

A total of 1,495 HCWs were included in the analysis. Of these, 42.8% (*n* = 637) were frontline HCWs (who had contact with COVID-19 patients), 23.1% (*n* = 344) were second-line HCWs (who only had contact with non-COVID-19 patients) and 34.1% (*n* = 508) had no contact with any patients. Participating hospitals offer various counseling and support services for their staff. The percentage of respondents who were unaware of available counseling and support services ranged from 5.0 to 42.0%. Depending on the type of counseling and support services, 23.0–53.6% of the respondents indicated that counseling and support services were provided but not used, while 1.7–11.6% indicated that, despite the need for them, such services were not available. HCWs’ overall satisfaction with the provided counseling and support services and their unmet support needs differed by patient contact: Frontline HCWs reported more unmet needs for counseling and support than second-line HCWs, while second-line HCWs reported more unmet needs than HCWs without patient contact.

**Conclusion:**

The results indicate that hospitals should make more efforts to inform HCWs about available counseling and support services. Hospitals could also create networks where HCWs could share information about the type and content of services and their experiences with various counseling and support services. These steps would enable hospitals to respond more quickly and effectively to the problems facing HCWs during pandemics.

## 1. Introduction

The SARS-CoV-2 pandemic is one of the biggest challenges facing healthcare systems worldwide. Due to the current situation, healthcare workers (HCWs) in particular are experiencing high levels of emotional and physical stress. Increased risk of infection at work, changes to work processes and working conditions, staff shortages, and private stressors such as the impacts of school and nursery closures on child care responsibilities are some of the challenges HCWs are currently facing (1–4). These difficulties can increase the stress of people working in the healthcare system (5). Numerous national and international studies have shown that both HCWs directly involved in caring for COVID-19 patients and second-line HCWs in non-epidemic departments (6) report work-related stress and symptoms of depression, anxiety or insomnia (7–16).

The well-being and health of HCWs are essential to pandemic response. Therefore, hospitals are trying to help personnel cope with stressful working conditions, especially during the pandemic. Services include daily updates on current information, telephone support/helplines, supervision and other support services, such as courses on relaxation techniques (17–20). The aim of the present study is to examine HCWs’ awareness and utilization of support services at university hospitals in Germany. It also aims to identify these HCWs’ unmet needs for support services. In this context, a question arises: Could a national organizational approach that bundles support services from diverse clinics and makes them available to all employees contribute to faster, more effective problem solving in the future? Employee assistance programs (EAPs) can provide free, confidential short-term mental health services for individuals, referrals for advanced treatment and other services, such as stress management training (21). Evidence from a systematic review suggests that EAPs save organizations money and increase the well-being of most employees (22). Furthermore, another study found that implementation of EAPs led to reductions in absenteeism and presenteeism (23).

The egePan Unimed and PREPARED projects are funded by the German Federal Ministry of Education and Research as part of the Network University Medicine (Netzwerk Universitätsmedizin, NUM) initiative. This network aims to assess and implement strategies to manage the challenges of the pandemic and to support HCW stability (24). The Employee Assistance Program for University Hospitals (EAP Unimed) is part of the egePan and PREPARED projects. In a first step, staff members at occupational health offices, hospital hygiene departments, and departments of psychosomatic medicine and psychiatry filled out an online questionnaire about available counseling and support services at their hospitals. In a second step, HCWs at German university hospitals that are participating in the NUM egePan Unimed project were surveyed. This paper presents the results of this second survey, which assesses the following factors:

- HCWs’ awareness and utilization of counseling and support services, including their awareness of the type and content of available services,
- HCWs’ unmet needs for counseling and support services during the SARS-CoV-2 pandemic, including the type and content of unmet needs, and
- Differences among frontline HCWs (who have contact with COVID-19 patients), second-line HCWs (who have contact only with non-COVID-19 patients) and HCWs without patient contact in terms of unmet needs for support and overall satisfaction with the type and content of available services.

## 2. Materials and methods

### 2.1. Study design and study population

Data were collected via a cross-sectional online survey (LimeSurvey). The survey was conducted in German. HCWs working in hospitals in Germany during January and June 2021 participated voluntarily and anonymously in a self-rated questionnaire. Participants’ informed consent was obtained at the beginning of the questionnaire.

### 2.2. Sampling and recruitment

A link to the online survey was distributed to university hospitals in the NUM network that are part of the egePan Unimed project. The hospitals were asked to inform all personnel about the study and to invite them to participate by sending an e-mail with information about the study or via a link to the survey sent via intranet. There were no exclusion criteria regarding the target group. The total number of individuals invited to participate in the survey is unknown.

### 2.3. Questionnaire and measurement

Data were collected via a self-administered questionnaire comprising 67 variables divided into four sections: (i) sociodemographic data, (ii) health and well-being, (iii) pandemic-related working conditions, and (iv) awareness and utilization of counseling and support services in the hospitals, including the type and content of available services and unmet needs for services during the pandemic. To answer the study questions, 12 (13 items including one conditional question) questions from section i (participants’ demographic characteristics) and section iv were evaluated (primary outcome variables): “How were counseling or support services provided at your hospital for staff dealing with the pandemic?”; “Please indicate whether and to what extent counseling and support services are provided on the following topics” and “What specific support services would you like to see?” Table 1 presents the issues of the questionnaire. Participants were characterized (secondary outcome variables) by gender, age group and professional group (e.g., doctor, nurse, administrative). Participants’ evaluations of available support services were included in the analysis to provide additional information about the subjective assessment (secondary outcome variable). The variables “patient contact” and “contact with COVID-19 patients” were used as confounder variables in the chi-squared test, the Kruskal–Wallis Test and the Bonferroni-corrected Mann–Whitney U test, which were used to assess differences among employees based on patient contact.

**Table 1:** Issues of the questionnaire.

Types of counseling and support services	
	Online Information (e.g. E-Mails, Intranet)
	Telephone consultations
	Personal consultations
	Online (self-help) services (e.g. health apps)
	Video consultations
Content of counseling and support services	
	Occupational health and safety measures/behavior rules
	Regular Covid-19 information
	Changes in working processes (e.g. access restrictions, changes in team organisation)
	Psychological problems (e.g. anxiety)
	Individual health support (e.g. coping with stress, relaxation)
	Workplace arrangement (e.g. because of home office)
	Health issues (e.g. previous illness, pregnancy)
	Addiction
	Family support (e.g. child care, caring for relatives)
	Measures/behavior of risk groups
	Financial problems
Overall satisfaction with counseling and support services	

To answer the survey questions, respondents could choose from the following options: “Was offered; I used it and it was helpful”; “Was offered; I used it, but it was not helpful”; “Was offered, but I did not use it”; “Was not offered but would have been helpful”; “Was not offered, but I would not have needed it” and “I do not know.” In addition, the statement “I am satisfied with the support services at my workplace” was used to evaluate respondents’ overall satisfaction; they could respond to this item using a 7-point Likert scale (1 = completely disagree, 7 = completely agree). Participants could also exit the questionnaire without answering all the questions. Surveys that did not include answers to all the questions included in the present analysis were excluded from the evaluation.

### 2.4. Ethics approval

Ethical approval for the study was provided by the ethics committee of the State Chamber of Medicine in Rhineland Palatinate (Clearance number 2021–15572).

### 2.5. Data preparation and analysis

Chi-square tests of independence were used to investigate the relationship between respondents’ unmet needs (indicated by the response “Was not offered but would have been helpful”) regarding type and content of counseling and support services and their contact with patients (categorized based on patient contact). All respondents were categorized as frontline HCWs, second-line HCWs or HCWs without any patient contact. For comparisons for which a chi-squared test of independence yielded a significant result, post-hoc analyses (multiple *z*-tests of proportions with a Bonferroni correction) were used to determine which groups differed from each other.

A Kruskal–Wallis test was used to compare different groups of HCWs’ overall satisfaction with support services. For post-hoc group comparisons, a Bonferroni-corrected Mann–Whitney U test was used (*p* < 0.017 is considered statistically significant). To measure effect size, r was calculated by dividing the z-value by the square root of the sample size (r = Z/√N). An r of 0.1 represents a small effect size, 0.3 represents a medium effect size and 0.5 represents a large effect size (25).

All statistical analyses were performed using SPSS version 23.5 and Excel for Windows 2016 (Version 1.5).

## 3. Results

A total of 3,212 individuals working in hospitals participated in the survey. Of these, 1,495 participants were included in the following analyses; 54% of the questionnaires were excluded from our analysis as they were incomplete.

Table 2 shows the HCWs’ demographic characteristics. Most respondents were female (*n* = 1,103, 74.3%) and nurses (*n* = 444, 29.7%). About one-third of respondents (*n* = 508, 34.1%) reported having no patient contact, while 981 (65.9%) respondents reported having patient contact. Of these, 637 (42.8%) were frontline HCWs (contact with COVID-19 patients), while 344 (23.1%) were second-line HCWs (contact with non-COVID-19 patients only).

**Table 2:** HCWs characteristics (*n* = 1,495).

Variable		Number	Percent
Gender			
	Male	382	25.7
	Female	1,103	74.3
Age			
	18–30 years	239	16.0
	31–40 years	403	27.0
	41–50 years	347	23.3
	51–60 years	399	26.7
	61–70 years	104	7.0
Professional group			
	Physician	186	12.4
	Nurse	444	29.7
	Administrative staff	330	22.1
	Medical (technical) assistant	228	15.3
	Other staff*	307	17.0
Contact with patients			
	Frontline HCW (contact with COVID-19 patients)	637	42.8
	Second-line HCW (contact with non-COVID-19 patients only)	344	23.1
	HCW without patient contact	508	34.1

Valid percentages are shown. The following values are missing: gender: *n* = 10; age: *n* = 3; contact with patients: *n* = 6. *Other staff: technical staff (*n* = 60), scientific staff (*n* = 120), other (*n* = 127).

### 3.1. Type of counseling and support services

Table 3 presents the descriptive results regarding the types of counseling and support services available. Most respondents (92.4%) stated that they had received online information about the COVID-19 pandemic from their employers. 61.6% of these respondents stated that these online information services were helpful; 23.0% did not use them. More than half of respondents (60.9%) indicated that telephone consultations were offered, and approximately half (53.1%) reported that they had access to personal consultation services. Approximately one-third of respondents stated that online (self-help) services (37.0%) and video consultation services (34.7%) were available.

**Table 3:** Types of counseling and support services.

	n	Offered (in %)			Not offered (in %)		I do not know (in %)
		Helpful	Not helpful	I did not use it	Would have been helpful	Would not have needed it	
Online information	1,288	61.6	7.8	23.0	1.7	0.9	5.0
Telephone consultation services	1,274	4.2	3.1	53.6	4.1	8.7	26.3
Personal consultation services	1,281	5.5	1.0	46.6	6.0	7.7	33.2
Online (self-help) services	1,272	8.4	3.8	24.8	11.6	17.1	34.4
Video consultation services	1,275	2.6	0.9	31.2	5.4	17.9	42.0

Respondents indicated that the following services were not available but would have been helpful (suggesting unmet needs): online (self-help) services (11.6%), personal consultation services (6.0%), video consultation services (5.4%), and telephone consultation services (4.1%). Apart from online information services (5.0% were not aware of these), 26.3–42% of respondents did not know whether these types of counseling and support services were provided by their hospital (Table 3).

### 3.2. Content of counseling and support services

The content of counseling and support services provided by hospitals during the pandemic is shown in Table 4. Most respondents (84.3%) reported the availability of services addressing occupational health and safety measures/behavior rules. Approximately 70% of respondents reported that, during the pandemic, they had regularly received information about COVID-19 (72.4%) and about changes to working processes (70.1%). In addition, 50–60% of the respondents reported services related to individual health support (59.8%), psychological problems (59.3%), workplace arrangements (e.g., questions about setting up the workplace because of home office; 56.3%) and health issues (50.8%). Almost 50% of respondents reported that available services included family support (48.3%), information regarding the measures and behaviors defining groups at high risk from COVID-19 (48.1%) and information on addiction (47.3%). 10.7% of the respondents reported services targeting financial problems.

**Table 4:** Content of counseling and support services.

	n	Offered (in %)			Not offered (in %)		I do not know (in %)
		Helpful	Not helpful	I did not use it	Would have been helpful	Would not have needed it	
Occupational health and safety measures/behavior rules	1,272	57.8	6.8	19.7	2.6	1.5	11.6
Regularly updated information about COVID-19	1,255	49.0	6.5	16.9	6.1	2.6	18.8
Changes to work processes	1,264	48.1	9.3	12.7	7.2	5.5	17.2
Individual health support	1,261	8.4	1.9	49.5	15.3	3.3	21.6
Psychological problems	1,257	2.4	1.5	55.4	8.4	4.9	27.4
Workplace arrangements	1,264	26.0	3.6	26.7	12.2	13.1	18.4
Health issues	1,263	8.2	2.5	40.1	9.1	5.9	34.3
Family support	1,262	3.7	1.1	43.5	12.0	6.2	33.5
Measures/behavior of risk groups	1,261	9.4	2.5	36.2	11.7	5.2	35.1
Addiction	1,254	1.3	0.9	45.1	3.6	7.7	41.5
Financial problems	1,257	0.9	0.3	9.5	8.1	15.6	65.6

Most of the unmet needs reported by respondents were related to individual health support services (15.3%), workplace arrangement services (12.2%), family support services (12.0%) and information services related to the measures or behaviors defining high-risk groups (11.7%). Some respondents indicated a need for more support around health issues (9.1%), psychological problems (8.4%), and financial problems (8.1%). In addition, 7.2% would have liked more support related to changes to work processes, and 6.1% would have preferred more regular updates on information related to COVID-19 (Table 4).

In total, 65.6% of the respondents reported that they were not aware of any support services related to financial problems. In addition, 33 to 41% of respondents did not know whether special services for addiction (41.5%), measures/behavior of risk groups (35.1%), family support (33.5%) or health issues (34.3%) were available. Around a quarter (27.4%) of the respondents were unaware of services addressing psychological problems, and 21.6% were not aware of individual health support services. In addition, 18.8% reported that they did not regularly receive information about COVID-19; 18.4% were not aware of support related to workplace arrangements, and 17.2% were not aware of support related to changes in working processes. Finally, 11.6% of the respondents were unaware of services related to occupational health and safety measures/behavior rules (Table 4).

Regarding the use and rating of the content of available counseling and support services (Table 4), most respondents reported that services related to occupational health and safety measures/behavior rules (57.8%), regularly updated information about COVID-19 (49.0%) and information about changes to work processes (48.1%) were helpful during the pandemic. Between 40 and 60% reported not using counseling and support services addressing individual health support (49.5%), psychological problems (55.4%), health issues (40.1%), family support (43.5%), measures/behaviors of risk groups (36.2%), and addiction (45.1%). Services related to measures/behavior of risk groups, health issues, individual health support, family support, psychological problems, addiction and financial problems were relatively rarely used. Few respondents indicated that services related to the measures/behavior defining risk groups (9.4%), health issues (8.2%), and individual health support (8.4%) were helpful. Services related to family support, psychological and financial problems were also rarely reported as helpful.

### 3.3. Unmet needs regarding type and content of counseling and support services based on patient contact

**Figure 1:**
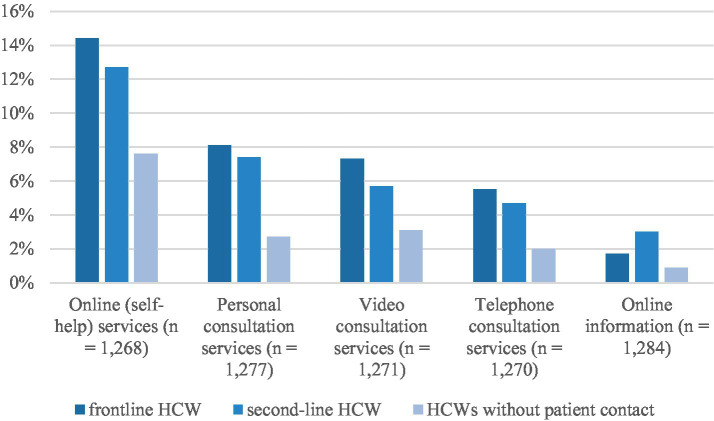
Unmet needs regarding type of counseling and support services.

There is a significant association between unfulfilled HCWs’ unmet needs and the type of patient contact they engage in Figure 1. Frontline and second-line HCWs expressed a greater need for online (self-help) services [X2 (2, *N* = 1,268) = 11.362, *p* = 0.003], personal consultation services [X2 (2, *N* = 1,277) = 14.162, *p* < 0.001], video consultation services [X2 (2, *N* = 1,271) = 8.062, *p* = 0.018] and telephone consultation services [X2 (2, *N* = 1,270) = 7.938, *p* = 0.019].

Post-hoc analyses showed that HCWs without patient contact rated personal consultation services differently to frontline and second-line HCWs. HCWs without patient contact also rated online (self-help) services, video consultation services and telephone consultation services differently to frontline HCWs. There was no significant difference between ratings by HCWs without patient contact and second-line HCWs, and there was no significant difference between ratings by frontline and second-line HCWs (Figure 1).

**Figure 2:**
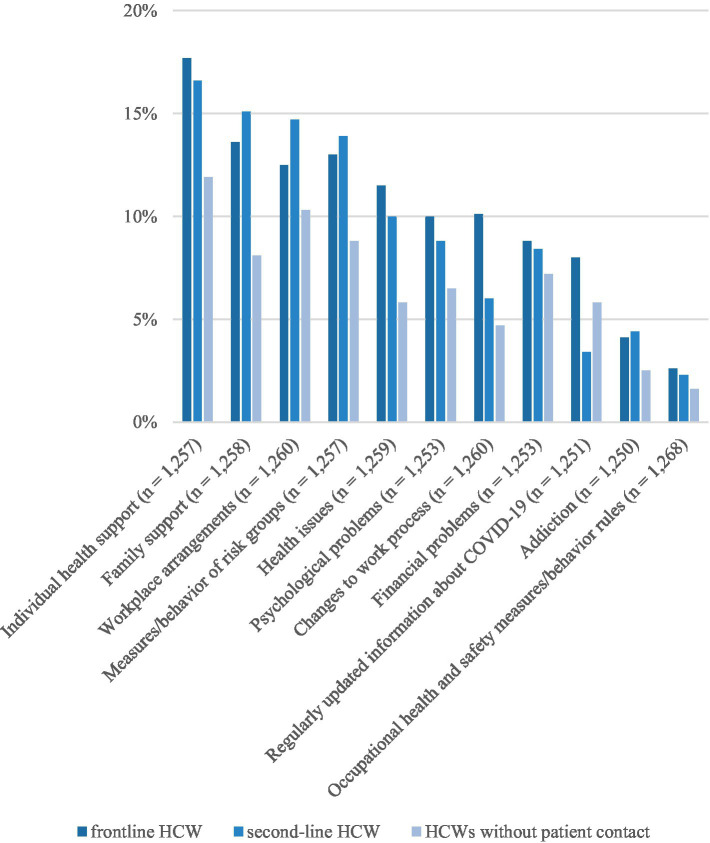
Unmet needs for counseling and support services.

In general, frontline and second-line HCWs reported more unmet needs for counseling and support services than HCWs without patient contact (Figure 2). HCWs without patient contact indicated significantly fewer unmet needs for the following types of services than frontline and second-line HCWs: individual health support [X2 (2, *N* = 1,257) = 6.719, *p* = 0.035], family support [X2 (2, *N* = 1,258) = 10.390, *p* < 0.006], health issues [X2 (2, *N* = 1,259) = 9.563, *p* = 0.008], changes to work processes [X2 (2, *N* = 1,260) = 11.224, *p* < 0.004] and regular information about COVID-19 [X2 (2, *N* = 1,251) = 7.030, *p* = 0.030]. HCWs with different types of patient contact did not indicate different unmet needs for services addressing occupational health and safety measures/behavior rules, workplace arrangements, measures/behavior of risk groups, financial problems, psychological problems or addiction.

Post-hoc analyses showed that frontline HCWs rated individual support services addressing health support, health issues and changes to work processes significantly differently to HCWs without patient contact. Furthermore, frontline and second-line HCWs expressed significantly more unmet needs for family support than HCWs without any patient contact. Regarding regular COVID-19 information services, there was a significant difference between second-line HCWs and HCWs without any patient contact as well as frontline HCWs (Figure 2).

### 3.4. Satisfaction with support services

The median value for respondents’ overall satisfaction with the support services offered by their employers was 5 on a scale from 1 (completely disagree) to 7 (completely agree). HCWs with different levels of patient contact indicated statistically significant differences in overall satisfaction with support services [χ^2^_(2)_ = 29.992, *p* < 0.001]. The mean rank satisfaction score for HCWs without any patient contact was 701.41; this score was 625.34 for second-line HCWs and 575.11 for frontline HCWs. The Bonferroni post-hoc test showed that frontline HCWs (median = 4) differed significantly to both second-line HCWs (median = 5, *z* = −1.981, *p* = 0.048, *r* = 0.07) and HCWs without any patient contact (median = 5, *z* = −5.425, *p* < 0.001, *r* = 0.17). There was also a significant difference between second-line HCWs and HCWs without any patient contact (*z* = −2.914, *p* = 0.004, *r* = 0.11).

## 4. Discussion

This paper presents the results of a survey of HCWs at German university hospitals.

The results of the study suggest that the included hospitals offered a range of counseling and support services for staff. However, several issues were identified:

- The percentage of respondents who were unaware of available counseling and support services varied but was sometimes as high as 66%.
- A high percentage of respondents (between 10 and 56%) were aware of the available counseling and support services but did not use them, while others (3–15%) reported a need for services that were not available at their hospitals.
- HCWs’ overall satisfaction with available counseling and support services differed according to patient contact (frontline HCWs were less satisfied than second-line HCWs and HCWs without patient contact).
- HCWs’ unmet needs for counseling and support services also differed according to patient contact (in general, frontline HCWs reported more unmet needs than second-line HCWs, and second-line HCWs reported more unmet needs than HCWs without patient contact). On this point, the differences between frontline HCWs and second-line HCWs were less pronounced than those between HCWs with patient contact (whether frontline or second-line) and HCWs without patient contact.

### 4.1. Lack of awareness

The figures regarding participants’ awareness of support services vary depending on the type and content of counseling and support services. However, with a few exceptions, 20–40% of the respondents were uninformed about available services. These results indicate that simply offering counseling and support services is not enough. These services must also be promoted via appropriate channels. Hospitals can inform HCWs about available services via e-mail or the intranet but also by using advertising posters or other marketing concepts.

### 4.2. (Missing) types and content of counseling and support services

Almost all respondents reported that they received online information about COVID-19 from their employers, and a large percentage indicated that this information was very helpful during the pandemic. About half of the respondents were aware of telephone and personal consultation services, and about a third were aware of online (self-help) and video consultation services. However, only 3–8% of the respondents used these types of services, so most respondents who were offered these services did not use them. At the same time, 4–12% of the respondents reported that no such services were available, even though they would have been helpful. These figures demonstrate that hospital networking could effectively provide all hospital staff with access to various support services. These findings also highlight the importance of online services; 8% of the respondents reported that online (self-help) services helped them meet the demands of their work, and almost 12% indicated that they would have found online services helpful. The results of the present study underpin the importance of online services, as they can provide sufficiently flexible support to HCWs. These findings also confirm recent developments, as various online resources for HCWs have been offered or developed during the pandemic (26).

There is no doubt that the pandemic has changed HCWs’ working processes and conditions (3, 4). Regarding the content of counseling and support services, the vast majority of the respondents indicated they received services related to occupational health and safety measures and behavior rules. Among those who reported receiving this information, most found this support helpful. However, only about 70% of the respondents reported that they regularly received information about COVID-19 and changes to work processes, while most respondents reported that this kind of information was helpful. Only about half of the respondents reported having access to services addressing individual health support, psychological problems, workplace arrangements, health issues, family support, addiction or the measures/behaviors of risk groups. However, apart from services regarding workplace arrangements, most respondents reported that they did not use these types of services.

In the present study, many respondents indicated that counseling and support services were available at their hospitals, but they did not use them. Meanwhile, other respondents indicated that they would have liked to have access to such services, but none were available at their hospitals. This suggests that an EAP program and hospital counseling and support service networks could effectively target and address employees’ stress. For example, if all hospitals shared access to counseling and support services and listed those services with a central entity, ideally online, HCWs could access support services at any hospital. A network of many clinics would also offer two other advantages. Firstly, underutilized support services would be available to employees of other hospitals who might need them, and secondly, individual hospitals could be relieved of the pressure to provide all types of support services for their employees.

### 4.3. Unmet needs depend on patient contact

Frontline and second-line HCWs’ unmet needs and overall satisfaction with the types and content of available counseling and support services differed significantly to those of HCWs without patient contact. Frontline and second-line HCWs reported a need for more types of counseling and support services and a broader range of counseling content than HCWs without patient contact. The two groups of HCWs with patient contact also differ: Frontline HCWs indicated more unmet needs than second-line HCWs. For overall satisfaction with counseling and support services, a similar picture emerges: Frontline and second-line HCWs were less satisfied with support services than HCWs without any patient contact. These findings are consistent with those of various studies around the world reporting that, during the pandemic, frontline HCWs in particular experienced increased stress, depression, anxiety and insomnia (16, 27). However, our findings indicate that, not only frontline HCWs, but also second-line HCWs and other hospital staff desire various types of counseling and support services. Therefore, future studies should not group second-line HCWs with HCWs without patient contact [see, e.g., (28)]. Furthermore, our results confirm the results of another study that future support services should take the particular needs of different groups of HCWs into account and offer tailored support (29).

### 4.4. Limitations

The method of sampling and recruitment adopted in this study and the high dropout rate comprise two limitations of the study. We do not know whether hospitals in the network informed their staff about the study. As a result, the sample is not representative, as it is limited to participants who received the link to the survey. Presumably, employees with access to the internet at work were more likely to participate. In addition, the questionnaire used in the present study included some non-validated items that were developed for this study. These items were valuable as they targeted issues that a standardized questionnaire could not. Despite the high number of participants (1,495), our study has a relatively low response rate, as the 25 largest university hospitals in Germany have a total of around 160,000 employees (30). The possibility of response bias is another limitation of this study, as HCWs who searched for counseling and support services during the pandemic may have been more likely to respond to the survey.

## 5. Conclusion

Based on the results of this survey, the authors conclude that communication about the type and content of available counseling and support services is of central importance. Furthermore, the creation of a hospital network and the development of a pooled online resource for HCWs could address some of the issues identified here. Such a website could provide information on the types and content of counseling and support services provided by individual hospitals. Ideally, these counseling and support services should be accessible to all staff at network hospitals. This network would be similar to an EAP (as described in the introduction) and could be an important tool and point of contact for HCWs seeking help and support.

## Data availability statement

The raw data supporting the conclusions of this article will be made available by the authors, without undue reservation.

## Ethics statement

The studies involving human participants were reviewed and approved by the Ethics Committee of the State Chamber of Medicine in Rhineland Palatinate (Clearance number 2021–15572). The patients/participants provided their written informed consent to participate in this study.

## Author contributions

LM, CI, PK, MT, HFW, NR, OT, KL, HW, SL, BM, SH, KA, SU, MB, and D-MR participated in the conception and design of the study and monitored data collection. ED analyzed the data and wrote the manuscript. ED, LM, CI, PK, MT, HFW, NR, OT, KL, HW, SL, BM, SH, KA, SU, MB, and D-MR participated in data interpretation, drafting, and revising the manuscript. All authors read and approved the final manuscript.

## Funding

egePan Unimed and PREPARED are funded by the German Federal Ministry of Education and Research as part of the Netzwerk Universitätsmedizin (NUM) initiative (Grant-No.: 01KX2021). The egePan project leads are Prof. Dr. Jochen Schmitt und Dr. Michael von Wagner. PREPARED project leads are Prof. Dr. Simone Scheithauer and Prof. Dr. Jochen Schmitt.

## Conflict of interest

The authors declare that the research was conducted in the absence of any commercial or financial relationships that could be construed as a potential conflict of interest.

## Publisher’s note

All claims expressed in this article are solely those of the authors and do not necessarily represent those of their affiliated organizations, or those of the publisher, the editors and the reviewers. Any product that may be evaluated in this article, or claim that may be made by its manufacturer, is not guaranteed or endorsed by the publisher.
